# Anaemia in Acute HIV-1 Subtype C Infection

**DOI:** 10.1371/journal.pone.0001626

**Published:** 2008-02-20

**Authors:** Koleka Mlisana, Sara C. Auld, Anneke Grobler, Francois van Loggerenberg, Carolyn Williamson, Itua Iriogbe, Magdalena E. Sobieszczyk, Salim S. Abdool Karim, for the CAPRISA Acute Infection Study Team

**Affiliations:** 1 Centre for the AIDS Programme of Research in South Africa (CAPRISA), University of KwaZulu-Natal, Durban, South Africa; 2 Columbia University, New York, New York, United States of America; 3 Institute for Infectious Diseases and Molecular Medicine, University of Cape Town, Cape Town, South Africa; University of California San Francisco, United States of America

## Abstract

**Background:**

The high prevalence of anaemia and the increased morbidity and mortality associated with anaemia during AIDS has been well described yet there has been little information about anaemia and changes in haemoglobin levels during acute and early HIV-1 infection.

**Methods:**

HIV-negative women (n = 245) were enrolled into an observational cohort as part of the Centre for the AIDS Programme of Research in South Africa (CAPRISA) Acute Infection Study. Acute infection was diagnosed following a positive HIV RNA PCR in the absence of antibodies, or detection of HIV-1 antibodies within 3 months of a previously negative antibody test. Haemotologic parameters were assessed before infection and at regular intervals in the first twelve months of HIV infection.

**Results:**

Fifty-seven participants with acute HIV infection were identified at a median of 14.5 days post-infection (range 10–81) and were enrolled in the CAPRISA Acute Infection cohort at a median of 41 days post-infection (range 15–104). Mean haemoglobin prior to HIV-1 infection was 12.7 g/dL, with a mean decline of 0.46 g/dL following infection. The prevalence of anaemia increased from 25.0% prior to HIV-1 infection to 52.6% at 3 months post-infection, 61.1% at 6 months post-infection, and 51.4% at 12 months post-infection.

**Conclusions:**

Haematologic derangements and anaemia with a trend towards iron deficiency are common with acute HIV-1 subtype C infection in this small cohort. The negative impact of anaemia concurrent with established HIV infection upon morbidity and mortality has been well documented but the prognostic potential and long-term effects of anaemia during acute HIV-1 infection remain unknown.

## Introduction

Anaemia has been well described in established HIV-1 infection, with an estimated prevalence ranging from 10% in asymptomatic HIV-infected patients to 92% in patients with AIDS[1], but there is little data on red blood cell parameters during the acute stages of HIV-1 infection. In established HIV infection, lower haemoglobin levels have been shown to correlate with decreasing CD4+ cell counts[2] and multiple studies have found an association between anaemia during established infection and a faster progression to AIDS and death [3]–[9]. Many of these studies may be limited in their applicability to developing countries as they were conducted in men with predominantly HIV-1 subtype B infection. However, a study from Tanzania found that among women with World Health Organization (WHO) clinical stage 1 or 2 disease, anaemia was associated with a more rapid decline in CD4 cell count and an increased mortality [10]. The high baseline rates of anaemia in many developing countries [11] together with a growing AIDS epidemic could further increase the burden of anaemia in these regions.

The aetiology of HIV-associated anaemia remains uncertain but appears to be multifactorial. Potential mechanisms include malnutrition, decreased haematopoietic cell production, diminished capacity of the haematopoietic stroma to respond to increased demand, and impaired erythropoietin feedback secondary to excess inflammatory cytokines[1], [12]–[15]. HIV-infected patients have been noted to have reduced levels of serum iron which would suggest iron-deficiency as the cause of the anaemia, however microcytosis is rarely seen in these patients [1].

While there is limited data about anaemia during acute HIV infection, it has been suggested that anaemia at the time of seroconversion may have implications for the clinical progression of the disease [16]. In a study of 42 seroconverters from Haiti, anaemia at the time of seroconversion was a predictor of rapid HIV disease progression.

The objective of this study was to describe the prevalence and characteristics of anaemia during the first 12 months following infection in a southern African population. We report on early haematologic changes in a cohort of 57 South African women with acute HIV-1 subtype C infection, twenty-three of whom had haematologic profiles available prior to infection.

## Materials and Methods

### Study Population

The Centre for the AIDS Programme of Research in South Africa (CAPRISA) Acute Infection Study is a prospective observational cohort study of reproductive age women that will provide information about the clinical, immunological, and virological natural history of HIV-1 subtype C infection. HIV-uninfected women at high risk of infection were recruited into an HIV-negative cohort where they received extensive risk reduction counseling including condom provision and underwent monthly HIV testing. Women from the cohort with evidence of new HIV-1 infection on monthly testing were enrolled into the acute HIV infection phase of the study. In addition, seroconverters were also identified from other studies on the basis of a positive HIV antibody test within 3 months of a negative test. Participants with acute infection were followed weekly for 3 weeks, fortnightly until 3 months, monthly until 1 year post-enrollment and every 3 months thereafter. Any participant with a CD4+ cell count less than 350 cells/mm^3^ at more than one study visit was referred to an antiretroviral treatment program; none of the women in this study took antiretrovirals at any time. Ethics approval for this study was obtained from the Nelson Mandela School of Medicine in Durban, the University of Cape Town, and the University of Witwatersrand in Johannesburg, South Africa. Written informed consent was obtained from all participants.

### Clinical Data and Laboratory Methods

Participants in the HIV-negative cohort underwent a baseline evaluation that included a physical examination and blood collection for a full haematological profile. Two HIV-1 rapid antibody tests (Determine: Abbott Laboratories, Tokyo, Japan and Capillus; Trinity Biotech, Jamestown, NY, USA) were performed on a monthly basis and pooled PCR testing (Amplicor v1.5: Roche Diagnostics, Rotkreuz, Switzerland) for HIV-1 RNA was performed on all antibody negative samples. All samples identified to be HIV-infected through the pooling assay were confirmed using a quantitative RNA PCR (Amplicor v1.5) and an HIV enzyme immunoassay (EIA) test (BEP 2000; Dade Behring, Marburg, Germany) on the same and subsequent samples from the participant.

Acute infection was diagnosed following a positive HIV RNA PCR in the absence of HIV-1 antibodies (i.e. pre-seroconversion) or detection of HIV-1 antibodies within 3 months of a previously negative antibody test. Time of infection was defined as 14 days prior to a positive RNA PCR assay in the absence of HIV-1 antibodies or as the mid-point between the last HIV seronegative test and the first HIV seropositive test.

Haemoglobin was measured using an Abbott Celldyne 3700 machine (Abbott Laboratories, Abbott Park, IL, USA) and anaemia was defined as a haemoglobin value of less than 12g/dL in keeping with the WHO definition[11]. Viral loads were measured using the Amplicor v1.5 machine.

All analyses were conducted using the SAS statistical package version 9.1 (SAS Institute, Cary, NC, U.S.A.). A paired t-test was used to determine differences between groups at different time points.

## Results

Between October 2004 and October 2006, 57 women with acute HIV-1 infection were identified; 28 from the HIV-negative cohort and 29 from other seroincidence studies. The mean age of the women in the acutely HIV-infected cohort was 27.3 years (standard deviation [SD] 8.70) and their mean body mass index was 28.5 kg/m^2 ^(SD 7.15). The women with acute infection were identified at a median of 14.5 days post-infection (range 10-81) and were enrolled into the Acute Infection cohort at a median of 41 days post-infection (range 15–104).

**Figure 1:**
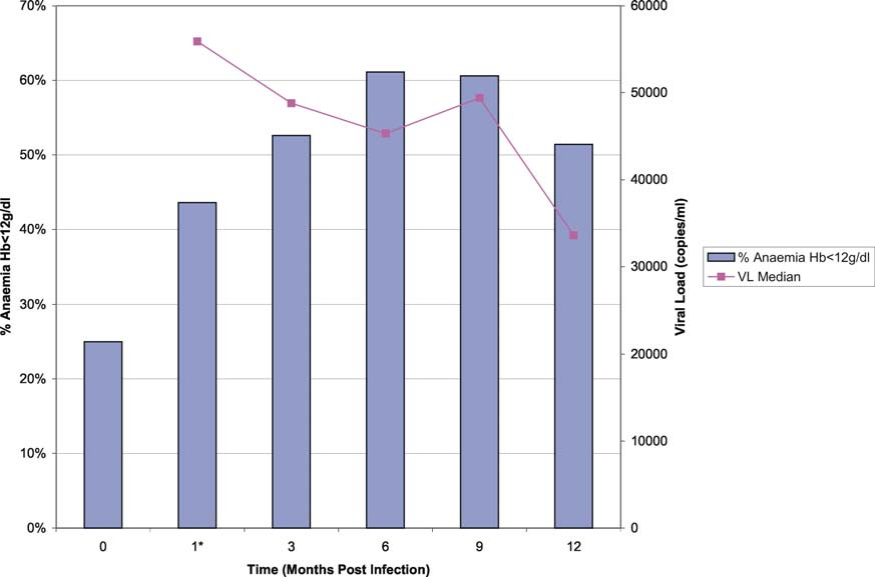
Prevalence of anaemia (Hb<12g/dL) prior to (time = 0) and in the months following acute HIV infection vs. median HIV viral load (copies/mL). *Note: month 1 represents the first post-infection visit which occurred at a median of 42 days post-infection (range 16-86).

For the 28 women from the HIV-negative cohort, the mean pre-infection haemoglobin level was 12.7 g/dL (SD 1.40) and eleven (39.3%) of them were anaemic; these results are summarized in Table 1. From pre-infection to the first post-infection study visit, the mean decline in haemoglobin was 0.46 g/dL (SD 1.09). The mean decline in haemoglobin between identification of acute infection and 3 months post-infection was 0.55 g/dL (SD 1.05) and between identification of acute infection and 6 months post-infection was 0.93 g/dL (SD 1.16). For the 26 participants with data available at 12 months, there was a mean increase of 0.10 g/dl (SD 0.97) between the 6 and 12 months post-infection measurements. The prevalence of anaemia, as defined by a haemoglobin level less than 12 g/dL, increased steadily throughout the first 6 months following acute infection, reaching 61.1% at 6 months, and remained close to 51.4% at 12 months post-infection (Figure 1).

**Table 1:** Haematologic parameters and median viral load prior to infection and by post-infection month.

	Pre-seroconversion (n = 28)	Post-seroconversion* (n = 55)	3 months (n = 57)	6 months (n = 36)	9 months (n = 33)	12 months (n = 35)
Mean (SD) haemoglobin (g/dl)	12.68 (1.40)	12.36 (1.35)	11.81 (1.51)	11.39 (1.57)	11.39 (1.62)	11.41 (1.41)
% (#) with Anaemia (Hb<12g/dl)	25.0% (7)	43.6% (24)	52.6% (30)	61.1% (1722)	60.6% (20)	51.4% (18)
Mean (SD) MCV (fl)	85.60 (6.64)	83.97 (6.20)	83.68 (6.41)	80.86 (7.23)	79.83 (8.38)	81.72 (7.94)
Mean (SD) MCH (pg)	29.13 (2.75)	28.32 (2.55)	28.27 (2.68)	26.72 (2.89)	26.22 (3.02)	26.81 (3.02)
Mean (SD) RDW (%)	15.03 (1.34)	16.40 (2.00)	16.03 (1.78)	16.31 (2.04)	17.16 (2.28)	17.18 (2.47)
Median (range) viral load (copies/mL)		75600 (547–5 510 000)	48 800 (269–1 390 000)	45 300 (<400–673 000)	49 400 (<400–494 000)	33 600 (<400–1 750 000)

Normal values are as follows: Hb 12.0–16.5 g/dl, MCV 76–99 fl, MCH 26–34 pg, RDW 11–16%. (*Note: The post-seroconversion visit refers to the first visit after identification of acute infection and was at a median of 41.5 days post-infection (range 15–104). Also, since acute infections were identified at different time points, not all participants have data at each month post-infection.)

The pre-HIV infection mean corpuscular volume (MCV) and mean corpuscular haemoglobin (MCH) were 85.60 fl and 29.13 pg and declined with acute HIV infection by 1.78 fl (SD 2.74) and 0.98 pg (SD 1.46) respectively. The MCV and MCH had mean declines of 2.53 fl (SD 4.33) and 1.68 pg (SD 1.72) between acute infection and 12 months post-infection respectively. Meanwhile, the red cell distribution width (RDW) increased by 1.70% (SD 2.16) with acute infection and then increased by 0.73% (SD 2.17) between acute infection and 12 months post-infection.

While the mean serum iron, vitamin B12, and folate did not change significantly at the time of acute infection, over the course of the first twelve months following infection, the mean iron level dropped to below the lower limit of normal (Table 2). The mean ferritin level increased marginally at the time of acute infection from 65.13 ng/mL prior to infection to 79.09 ng/mL with acute infection. The mean ferritin then declined steadily to 41.14 ng/mL at 6 months, and 38.09 ng/mL at 12 months (p = 0.0006 and p = 0.0165 compared with seroconversion, respectively).

**Table 2:** Iron studies.

	pre-infection (n = 28)	acute infection (n = 57)	6 months post-infection (n = 43)	12 months post-infection (n = 37)
mean (SD) Fe (µmol/L)	10.79 (5.82)	9.64 (5.56)	7.93 (4.62)	7.58 (6.10)
mean (SD) B12 (pmol/L)	319.00 (105.08)	284.84 (164.07)	288.16 (141.4)	285.59 (129.07)
mean (SD) folate (nmol/L)	21.66 (10.73)	20.19 (8.08)	21.27 (7.29)	23.00 (10.09)
mean (SD) ferritin (ng/mL)	65.13 (55.82)	79.09 (87.73)	41.41 (42.97)	38.09 (42.35)

Normal ranges: Fe 9.0–30.4 µmol/L, B12 132–857 pmol/L, Folate >12.19 nmol/L, ferritin 20–300 ng/mL.

To determine if anaemia was correlated to disease progression, the relationship between viral load and haemoglobin level were compared at various intervals using Pearson's correlation coefficient. The Pearson's correlation coefficient between the haemoglobin value at 3 months and the viral load closest to the time of HIV infection was significant at –0.2731 (p = 0.0398). The correlations between haemoglobin level closest to the time of infection and haemoglobin rate of decline and the viral load at various time points (closest to time of infection, 6 months post-infection, and 12 months post-infection) were not significant.

Fifteen of the 57 women (26.3%) were anaemic at all of their follow-up visits. When comparing this subset of women with the rest of the group, there were no significant differences in their age, body-mass index, CD4 cell count (either at the first post-infection visit or the mean of all available visits), or median viral load. As would be expected, the mean MCV (75.8 vs. 84.5 fl, p = <0.0001) and the mean MCH (25.1 vs. 28.2 pg, p = <0.0001) were significantly lower for the women anaemic at every follow-up visit and the RDW (18.3% vs. 16.0%%, p = <0.0001) was significantly higher for the women anaemic at every follow-up visit. The mean ferritin was lower (35.4 vs. 61.2 ng/mL, p = 0.0107) for the women anaemic at every visit as well.

## Discussion

This study demonstrated a decline in haemoglobin levels following HIV-1 subtype C infection and a rise in the prevalence of anaemia from 25.0% prior to HIV-1 infection to 52.6% at 3 months post-infection, 61.1% at 6 months post-infection, and 51.4% at 12 months post-infection. The haemoglobin declined by 0.46 g/dL at the time of acute infection, by 0.93 g/dL during the first 6 months following infection, and then by a mean of just 0.10 g/dL in the subsequent 6 months. The mean haemoglobin was lowest and the prevalence of anaemia was highest at 6 months post-infection. In the following 6 months, the haemoglobin level remained low and did not recover to pre-infection levels. This observation may reflect that perturbations of haematopoiesis or of the red cell life cycle are occurring early in the course of HIV infection and that these parameters begin to stabilize at 6 months post-infection. Unfortunately, reticulocyte counts were not obtained and so we can only infer the haematopoietic response from the available parameters.

The anaemia of these participants trended towards microcytosis and hypochromia and this trend persisted throughout the first 12 months following HIV-1 infection. The trend towards microcytosis combined with the progressive decline in serum iron levels and the elevated red cell distribution width (RDW) point towards iron-deficiency as the most likely etiology of the anemia in this cohort. Although the slight elevations in serum ferritin in this cohort do not correspond with the typical profile of iron-deficiency, ferritin is an unreliable indicator of underlying iron-deficiency in the setting of acute infection.

Anaemia seen this early following acute infection could be thought to reflect impaired haematopoiesis in the setting of early, uncontrolled viral replication and an excess of inflammatory cytokines[17], as shown by our results at the time closest to HIV infection. There was no significant correlation between haemoglobin level or rate of haemoglobin decline and viral load at 6 or 12 months post-infection.

Previous reports have suggested that there is an increased risk of anaemia with more advanced HIV infection[5], however, here we demonstrate that anaemia occurs with increasing frequency during the early stages of HIV-1 infection. Whether this acute infection associated anaemia will resolve during the typically latent period prior to clinical AIDS remains to be seen. Furthermore, whether the presence or severity of anaemia during acute HIV-1 infection will predict anaemia in chronic infection and/or more rapid disease progression also remains to be seen. Given the results of Deschamps and colleagues in Haiti that anaemia at seroconversion predicted a four-fold more rapid progression to AIDS[16] and the results of other studies that have found anaemia at any point in HIV infection to be an independent risk factor for decreased survival [18], anaemia is a potentially early indicator of more rapid clinical course and could factor into clinical algorithms regarding antiretroviral initiation, particularly in resource constrained settings. Additionally, in light of the propensity for certain antiretroviral medications such as Nevirapine and Zidovudine (AZT) to exacerbate underlying anaemia, a high prevalence of anaemia in patients awaiting therapy could factor into decisions regarding medication choice.

It will be important to confirm these results in other cohorts and to continue to characterize the relationship between anaemia and clinical course of HIV-1 infection as this cohort progresses into established infection. Additionally, since this cohort was comprised entirely of women, it is unknown if these same patterns will hold true for men. Although since women are at greater baseline risk for anaemia than men, it is important to characterize the increased burden that HIV-infected women will face.

Anaemia is a common problem among South African women and, according to our results, becomes more common with early HIV-1 infection. The increasing prevalence of anaemia in this population during early HIV-1 infection is concerning in light of the detrimental impact of low haemoglobin on energy and physical functioning [19]. Further prospective data is necessary to define the significance of these findings and to determine the impact of anaemia in acute infection on subsequent disease progression. The ongoing CAPRISA Acute Infection study will continue to follow the cohort described in this report to investigate whether anaemia during acute HIV-1 infection has any long-term consequences.

## References

[1] Kreuzer KA, Rockstroh JK (1997). Pathogenesis and pathophysiology of anemia in HIV infection.. Ann Hematol.

[2] Savarino A, Pescarmona GP, Boelaert JR (1999). Iron metabolism and HIV infection: reciprocal interactions with potentially harmful consequences?. Cell Biochem Funct.

[3] Lau B, Gange SJ, Phair JP, Riddler SA, Detels R (2005). Use of total lymphocyte count and hemoglobin concentration for monitoring progression of HIV infection.. J Acquir Immune Defic Syndr.

[4] Moore R (1999). Human immunodeficiency virus infection, anemia, and survival.. Clin Inf Dis.

[5] Semba RD, Shah N, Klein RS, Mayer KH, Schuman P (2002). Prevalence and cumulative incidence of and risk factors for anemia in a multicenter cohort study of human immunodeficiency virus-infected and -uninfected women.. Clin Inf Dis.

[6] Lundgren JD, Mocroft A (2003). Anemia and survival in human immunodeficiency virus.. Clin Inf Dis.

[7] Mocroft A, Kirk O, Barton SE, Dietrich M, Proenca R (1999). Anaemia is an independent predictive marker for clinical prognosis in HIV-infected patients from across Europe.. AIDS.

[8] Sullivan PS, Hanson DL, Chu SY, Jones JL, Ward JW (1998). Epidemiology of anemia in human immunodeficiency virus (HIV)-infected persons: results from the multistate adult and adolescent spectrum of HIV disease surveillance project.. Blood.

[9] Zhou J, Kumarasamy N, TREAT Asia HIV Observational Database (2005). Predicting short-term disease progression among HIV-infected patients in Asia and the Pacific region: preliminary results from the TREAT Asia HIV Observational Database (TAHOD).. HIV Medicine.

[10] O'Brien ME, Kupka R, Msamanga GI, Saathoff E, Hunter DJ (2005). Anemia is an independent predictor of mortality and immunologic progression of disease among women with HIV in Tanzania.. J Acquir Immune Defic Syndr.

[11] WHO (1992). The Prevalence of Anaemia in Women: A Tabulation of Available Information. in Document WHO/MCH/MSM/92.2..

[12] Bahner I, Kearns K, Coutinho S, Leonard EH, Kohn DB (1997). Infection of human marrow stroma by human immunodeficiency virus-1 (HIV-1) is both required and sufficient for HIV-1 induced hematopoietic suppression in vitro: demonstration by gene modification of primary human stroma.. Blood.

[13] Kreuzer KA, Rockstroh JK, Jelkmann W, Theisen A, Spengler U (1997). Inadequate erythropoietin response to anaemia in HIV patients: relationship to serum levels of tumour necrosis factor-alpha, interleukin-6 and their soluble receptors.. British J Hematol.

[14] Moses AV, Williams S, Heneveld ML, Strussenberg J, Rarick M (1996). Human immunodeficiency virus infection of bone marrow endothelium reduces induction of stromal hematopoietic growth factors.. Blood.

[15] Moses A, Nelson J, Bagby GC (1998). The influence of human immunodeficiency virus-1 on hematopoeisis.. Blood.

[16] Deschamps M, Fitzgerald DW, Pape JW, Johnson WD (2000). HIV infection in Haiti: natural history and disease progression.. AIDS.

[17] Lawn SD, Butera ST, Folks TM (2001). Contribution of immune activation to the pathogenesis and transmission of human immunodeficiency virus type 1 infection.. Clin Microbiol Reviews.

[18] Berhane K, Karim R, Cohen MH, Masri-Lavine L, Young M (2004). Impact of highly active antiretroviral therapy on anemia and relationship between anemia and survival in a large cohort of HIV-infected women: Women's Interagency HIV Study.. J Acquir Immune Defic Syndr.

[19] Semba RD, Martin BK, Kempen JH, Thorne JE, Wu AW (2005). The impact of anemia on energy and physical functioning in individuals with AIDS.. Arch Intern Med.

